# Re-irradiation of recurrent vertebral metastasis after two previous spinal cord irradiation: A case report

**DOI:** 10.3389/fonc.2022.995376

**Published:** 2023-01-05

**Authors:** Rita Bentahila, Rémy Kinj, Constance Huck, Yasmine El Houat, Ange Mampuya, Constantin Tuleasca, Mahmut Ozsahin, Jean Bourhis, Luis Schiappacasse

**Affiliations:** ^1^ Department of Radiation Oncology, Lausanne University Hospital (CHUV), Lausanne, Switzerland; ^2^ Department of Neurosurgery, Lausanne University Hospital (CHUV), Lausanne, Switzerland

**Keywords:** retreatment, spine, stereotactic radiation, vertebral metastases, radiosurgery

## Abstract

**Background:**

Management of a recurrent vertebral metastasis in a situation of previously irradiated spinal cord is a challenging clinical dilemma.

**Case presentation:**

We report a first case of second retreatment of a spinal metastasis initially irradiated with standard radiotherapy and stereotactic body radiation therapy (SBRT), who subsequently progressed with imaging-confirmed local tumor progression at the same level. After a third course of irradiation with SBRT, a complete response was achieved. After 8 months of follow-up, the patients remain free of local recurrence.

**Conclusion:**

A third course of vertebral irradiation for a recurrent vertebral metastasis failing to two previous irradiations, in this particular case, have shown the feasibility and efficacy of the technique as a salvage treatment option. This approach could be used in a selected group of patient if an adequate dose is delivered to the target while observing critical tissue tolerance limits.

## Introduction

In recent years, the development of Stereotactic Body Radiotherapy (SBRT) for spinal metastases is emerging as a safe and effective ablative treatment for recurrent tumors. Modern prospective series and randomized trials have shown promising results on local control and pain relief of bone metastases (1, 2).

However, in the particular case of vertebral reirradiation, there have been concerns about spinal cord toxicity when treating recurrent metastases after conventional palliative radiotherapy or a first course of SBRT.

There are some reports about the safety and efficacy of SBRT in previously irradiated vertebral metastases (3–7), but to our knowledge, no report have been published for a patient treated several times.

In this report, we describe our experience with a single patient receiving a third irradiation for a T8 vertebral metastasis secondary to an invasive ductal carcinoma 15 years after the first conventional (2D) irradiation and 3 years after a first SBRT over the same lesion.

## Case presentation

We herein report the case of a women born in 1962 who was diagnosed with an invasive ductal carcinoma pT1b (0.9 cm) pN1a (2/17) cM0, grade 2, ER-positive and HER-2 positive in 1998. She underwent a right breast-conserving surgery and axillary lymph nodes dissection. After surgery, she received adjuvant treatment including chemotherapy, whole breast radiotherapy (including internal mammary chain (IMC) irradiation) and endocrine therapy by tamoxifen.

During the follow-up in 2006, a Fluoro-2-deoxy-D-glucose (FDG) PET-CT scan revealed an oligoprogression in the form of a regional submammary nodule and two bone metastases (T8 and left iliac wing). A biopsy confirmed the diagnosis of invasive ductal carcinoma grade 2, ER-positive and HER-2 positive.

**Figure 1:**
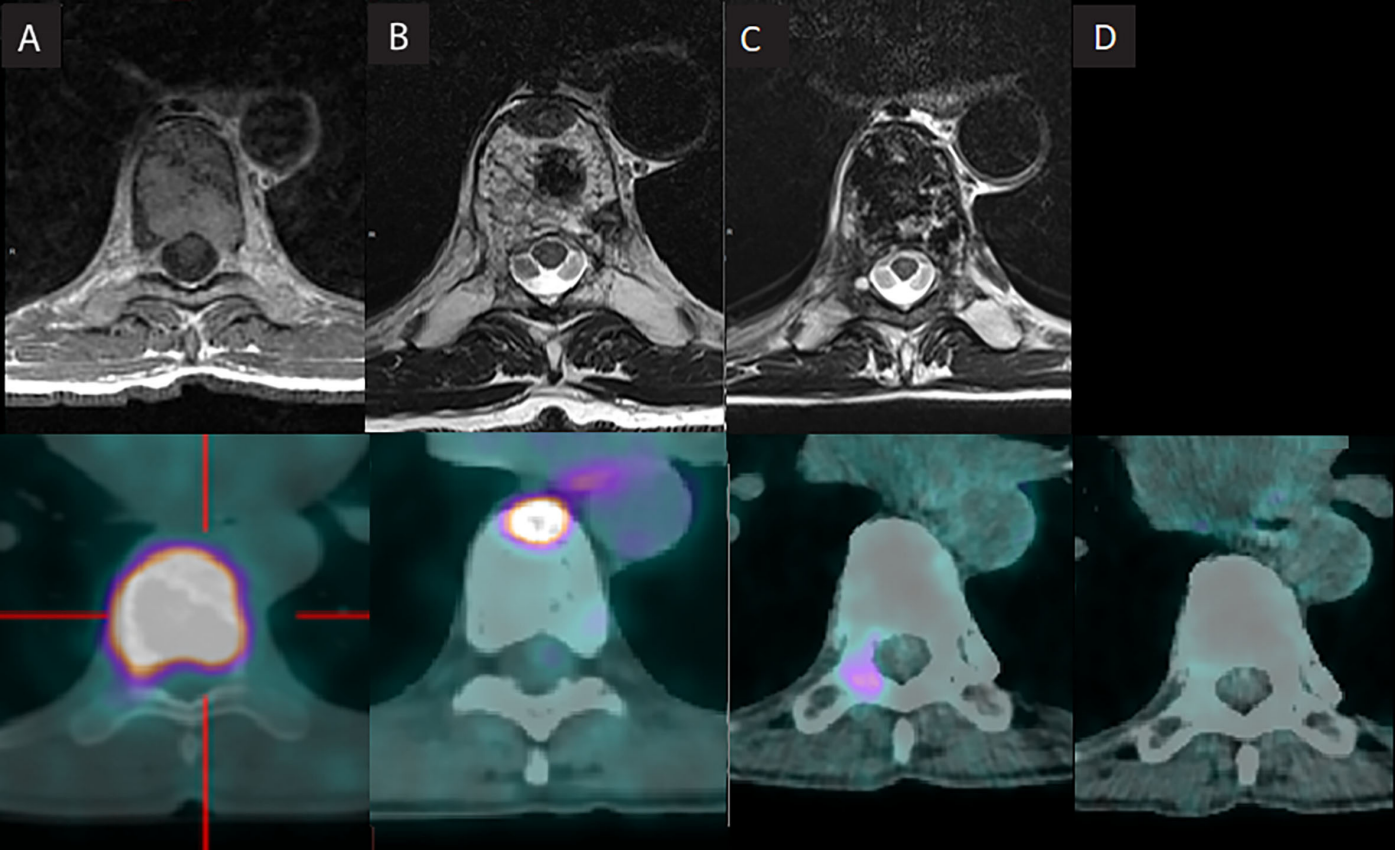
**(A–C)** T8 metastasis evolution after iterative irradiation. **(A)** 2006: First presentation: large deposit in the D8 vertebra with wedge compression (Bilsky grade 1a with epidural impingement). **(B)** 2014: First recurrence: osseous lysis lesion of the anterior portion of the T8 vertebra body **(C)**. 2021: Further recurrence: right transverse pedicle of D8 vertebra **(D)**. 2022: Recent follow-up (April 2022) showing complete response.

Spine MRI showed a large lytic metastasis of the T8 vertebra with wedge compression (Bilsky grade 1a with epidural impingement) (**Figure 1A**) (8).

**Figure 2:**
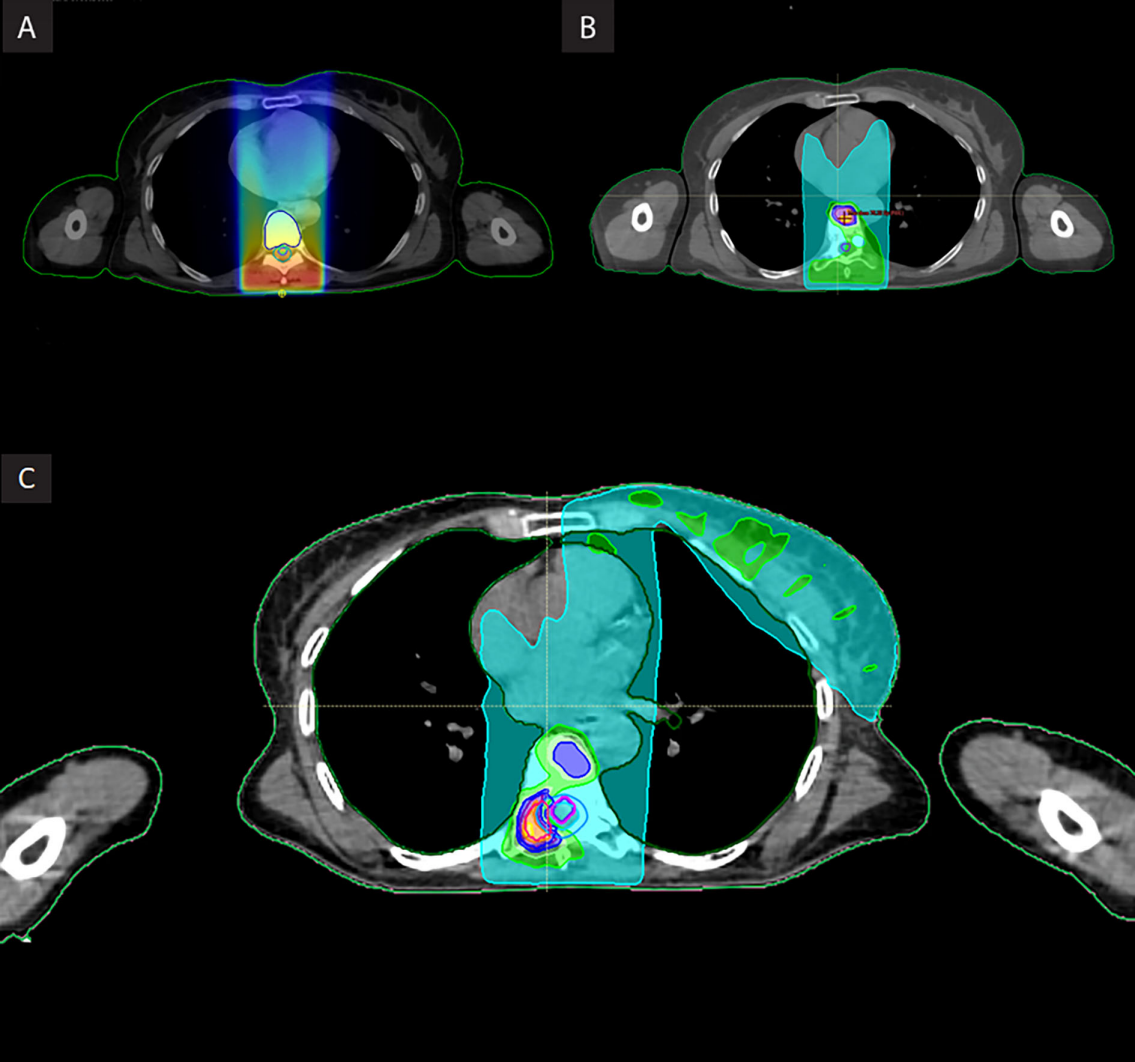
**(A–C)** Patient’s planning image with cumulative dose distribution. **(A)**. First course of 2D radiotherapy (30 Gy in 12 fractions) **(B)**. First re-irradiation vertebral SBRT (18 Gy in 3 fractions) **(C)**. Composite dose including vertebral after second SBRT (30 Gy in 5 fractions).

The patient received a conventional (2D) radiotherapy to the T7-T9 spine delivering a dose 30 Gy in 12 fractions of 2.5 Gy in April 2006, using a Siemens Primus linear accelerator system (Siemens, Concord CA, US). Patients planning images with dose distribution are shown in **Figure 2A**.

She started a systemic therapy with Leuprorelin acetate, Letrozole, and Ibandronate until January 2014.

In July 2014, evaluation with MRI and (FDG) PET-CT imaging revealed an osteolytic lesion of the anterior portion of the T8 vertebra body, in the previous treated field, suspect of local progression (**Figure 1B**).

A new biopsy of the T8 vertebra confirmed the recurrence of invasive ductal carcinoma. This was followed by a vertebral body cementoplasty on September 2014.

Spine surgery evaluation was not performed because, as shown by the radiotherapy scheme chosen in 2006, at that time the patient was considered a strictly palliative case, being managed as such outside from our institution.

A re-irradiation of the previously described T8 lesion using SBRT with a dose of 18 Gy in 3 fractions at isodose 80% was performed; the irradiation volume only included hypercaptation on PET-CT (**Figure 2B**). A CyberKnife robotic stereotactic radiotherapy system (Accuray, Sunnyvale, Concord CA, US) was used. This treatment was delivered using the Xsight spine tracking system, which co-relates imaging using bony anatomy for continuous imaging, repositioning, and tracking without fiducials, allowing a tracking of the target in real time. Doses were calculated using Precision Treatment Planning system (Accuray, Sunnyvale, Concord CA, US).

Following SBRT, the patient resumed systemic therapy with Trastuzumab and endocrine therapy until July 2015.

From July 2015 to January 2021, evaluation with FDG PET-CT imaging revealed oligoprogressive disease with bone lesions (C1, T6 and T8 vertebra, right acetabulum, left clavicle and left iliac wing), confirmed by biopsy. These lesions were treated with several lines of systemic therapy and SBRT over all lesions (with good metabolic response) except T8.

The T8 vertebra metastasis relapsed on right transverse pedicle and was treated using radiofrequency ablation in April 2018 (with complete response) and three more times in 2021, after new recurrences, obtaining only partial responses.

In October 2021, despite iterative radiofrequency treatments follow up with (FDG) PET-CT scan showed a new local progression on the right transverse pedicle of T8 vertebra (**Figure 1C**).

Spine surgery team evaluated the patient, and confirmed that surgery was not an option due to the technical challenge and the important risks inherent to this procedure.

Considering the short time between local recurrences after radiofrequency ablation, and after a dosimetric evaluation considering all radiation treatments impacting T8, a new SBRT was decided.

Before the last SBRT was performed, it was explained to the patient that there was a risk of radiculitis at the D9 level, due to the impossibility of respecting the tolerance dose of this nerve root, because of the previous delivered radiation.

A dose of 30 Gy in 5 fractions at isodose 80% (maximum dose in the PTV: 37.5 Gy.) was planned for the second course of SBRT (third course of irradiation) using a CyberKnife system (**Figure 2C**). This scheme is based on the one used at MD Anderson Cancer Center and published by Chang et al. in 2007.

Dose limits to the critical neural tissues (CNT) was determined using Sahgal et al. re-irradiation recommendations (9), and the report of the AAPM Task Group 101 on SBRT. For the calculations considering the spinal cord we used an α/β of 2; for the rest of OAR, an α/β of 3. Velocity AI software (Varian Medical Systems, Inc., Palo Alto, CA, US) was used to convert the different schemes and different fractionations to the equivalent in EQD2, giving an α/β ratio for the spinal cord of 2. We then performed a summation of all recalculated schemes in EQD2, using a rigid fusion and a ROI involving the region of the spine to be reirradiated. Then we calculated the residual safety margin (or dose limit) at the spinal cord level and other OARs, which we used to plan the treatment without exceeding the previously described constraints.

Technical characteristics of plans and dose parameters for all radiation treatments are shown in **Table 1**.

**Table 1:** Technical characteristics of plans and dose parameters for all radiation treatments.

Parameters	2D-conventional radiotherapy(1rst course)*	Re-irradiation SBRT(2nd course)	Re-irradiation SBRT(3rd course)	Cumulative dose
*Dose fractionation [Gy]*	30 Gy in 12 fractions	18 Gy in 3 fractions	30 Gy in 5 fractions	
*Technique*	2D radiotherapy	SBRT	SBRT	2D + SBRT
*PTV volume [cm^3^]*	N/A	2.76	5.87	
*PTV D95%*	N/A	19.02 Gy	23.43 Gy	
*PTV D50%*	N/A	20.16 Gy	33.88 Gy	
*Organs at risk (OARs)* *Spinal cord* *D_max_ EQD_2_ [Gy]*	34.7	2.89	3.94	42.78

Gy — Gray; cm^3^ — cubic centimetre; D_x_ cm^3^ — dose received by × cm^3^ of volume; D_max_ — maximum dose; EQD_2_ — equivalent dose in 2 Gy.

*We have included the dose delivered during the IMC irradiation (Dmax 5.46 Gy, corresponding to an EQD_2_ of 3.05 Gy). NA, not available.

After calculation of a composite dose in equivalent total doses in 2-Gy fractions (EQD_2_) of all the previous described treatments (including the IMC field dose contribution) spinal cord received a maximum dose (Dmax, 0.035 cc) of 42.78 Gy.

Patient completed re-irradiation SBRT without any side effect.

Following this treatment with SBRT re-irradiation, the patient resumed systemic treatment with Trastuzumab and Emtansine.

At 8 months of follow-up after the third course of irradiation, most recent assessment with (FDG) PET-CT scan shows complete response of the T8 vertebra metastasis (**Figure 1D**).

No evidence of toxicity secondary to the third course of irradiation according to CTCAE v.5 scale has been observed.

## Discussion

Treating vertebral metastases in the context of a previously irradiated spinal cord is a challenging clinical dilemma. Vertebras are a complex site for SBRT due to the proximity of the spinal cord. Risk of myelopathy and potential toxicity of progressive tumors must be carefully balanced by the radiation oncologist when evaluating the feasibility of a radiotherapeutic approach.

The most common site of distant breast metastases, occurring in around 40 to 51% of metastatic patients, is bone (10). An increment in the use of new imaging technologies and (FDG) PET-CT during the follow-up might be responsible for the increased incidence in diagnosis of isolated bone metastasis.

Moreover, in a context of prolonged survival due to new systemic and focal treatments, vertebral recurrences incidence tend to increase and their management becomes more challenging.

For a second course irradiation, often with 30 Gy in 5 fractions, most retrospective studies exhibited consistent results in terms of pain relief and sustained local control (3–7).

In a cohort of 59 patients, Garg et al. conducted a study on a single re-irradiation after spinal SBRT. The 1-year radiographic local control and overall survival for all patients was 76% (4). In a retrospective study, Thibault et al. concluded that a salvage second-course vertebral SBRT is feasible and efficacious after in-field failure of the first course of SBRT for spinal metastases. The median time to failure after the first course of SBRT was 11.7 months (7).

We report a first case of reirradiation of a vertebral recurrence after two previous courses of radiotherapy.

Time between the first irradiation and the second was 15 years, and 3 years between the second and third irradiation over the same lesion.

The time interval between two courses of radiation is not yet validated as a protective factor for toxicity. Therefore, the time-dependent recovery of neurological function and cumulative spinal cord dose limits remains largely hypothetical. Sahgal et al. suggests that SBRT given at least 5 months after conventional palliative radiotherapy appears to be safe, if several conditions are met (9).

At the time of last T8 progression, the patient presented a low burden volume of metastatic disease.

The recurrences were located in different parts of the T8 vertebra. Failure might be cause by insufficient extension of the radiation field beyond the visible tumor (not including pedicles and posteriors elements) or underdosed epidural space in order to limit spinal cord dose (11–13).

Dmax over the spinal cord was still not reached during two previous irradiations (conventional radiotherapy and SBRT) and distance between the targeted lesion (T8) and the spinal cord allowed us to avoid this critical organ, delivering radiation safely and respecting spinal cord constraints.

Following the previous described data, and on the basis of the published literature by Nieder et al. that described reirradiation spinal cord tolerances (14), a third course of radiation was decided.

With the support of our spinal surgery team, we have decided to accept the risk of radiculitis at the D9 level in order to avoid potential spinal cord threat due to the lack of control of this metastatic lesion. In the event of this complication, a surgical procedure would allow desensitization of this root, with only sensory consequences at the level of this dermatome for the patient.

A local complete response was achieved. After 6 months of follow-up, the patient remains without local recurrence and asymptomatic.

The benefits of SBRT, besides of local control, extend to the possibility of delaying the start of a new line of systemic treatment.

This case is the first case report on re-irradiating a vertebra after a conventional radiotherapy and SBRT previous courses of radiotherapy.

## Conclusion

Re-irradiation after standard irradiation and vertebral SBRT appears to be feasible with an acceptable level of toxicity, and can be considered as an efficacious salvage treatment option if delivered to a selected group of patients with an adequate dose delivered to the target while observing critical neural tissue tolerance limits.

Even if previously described retrospective series suggest the efficacy and safety of vertebral re-irradiation using SBRT, further evidence is needed before spreading the use of this technique in an extreme situation like ours.

## Data availability statement

The raw data supporting the conclusions of this article will be made available by the authors, without undue reservation.

## Ethics statement

Ethical review and approval was not required for the study on human participants in accordance with the local legislation and institutional requirements. The patients/participants provided their written informed consent to participate in this study. Written informed consent was obtained from the individual(s) for the publication of any potentially identifiable images or data included in this article.

## Author contributions

Conceptualization, RB and LS; methodology, RB and LS; validation, RK, AM, CT, MO, JB and LS; data curation, RB, CH and YH; writing—original draft preparation, RB, RK, CH, YH, and LS; writing—review and editing, RB, RK, CH, YH, AM, CT, MO, JB and LS; supervision, LS; project administration, LS. All authors contributed to the article and approved the submitted version.
