# Erratum: Re-irradiation of recurrent vertebral metastasis after two previous spinal cord irradiation: A case report

**DOI:** 10.3389/fonc.2023.1170270

**Published:** 2023-03-01

**Authors:** Frontiers Production Office

**Affiliations:** Frontiers Media SA, Lausanne, Switzerland

**Keywords:** retreatment, spine, stereotactic radiation, vertebral metastases, radiosurgery

An omission to the funding section of the original article was made in error. The following sentence has been added: “Open access funding was provided by the University of Lausanne”.

The publisher apologizes for this mistake.

The original version of this article has been updated.

